# Large-scale molecular dynamics simulation of coupled dynamics of flow and glycocalyx: towards understanding atomic events on an endothelial cell surface

**DOI:** 10.1098/rsif.2017.0780

**Published:** 2017-12-06

**Authors:** Xi Zhuo Jiang, Haipeng Gong, Kai Hong Luo, Yiannis Ventikos

**Affiliations:** 1 Department of Mechanical Engineering, University College London, Torrington Place, London WC1E 7JE, UK; 2 MOE Key Laboratory of Bioinformatics, School of Life Sciences, Tsinghua University, Beijing 100084, People's Republic of China

**Keywords:** molecular dynamics, coupled dynamics, flow shear stress, glycocalyx, endothelial cells

## Abstract

The glycocalyx has a prominent role in orchestrating multiple biological processes occurring at the plasma membrane. In this paper, an all-atom flow/glycocalyx system is constructed with the bulk flow velocity in the physiologically relevant ranges for the first time. The system is simulated by molecular dynamics using 5.8 million atoms. Flow dynamics and statistics in the presence of the glycocalyx are presented and discussed. Complex dynamic behaviours of the glycocalyx, particularly the sugar chains, are observed in response to blood flow. In turn, the motion of the glycocalyx, including swing and swirling, disturbs the flow by altering the velocity profiles and modifying the vorticity distributions. As a result, the initially one-dimensional forcing is spread to all directions in the region near the endothelial cell surface. Furthermore, the coupled dynamics exist not only between the flow and the glycocalyx but also within the glycocalyx molecular constituents. Shear stress distributions between one-dimer and three-dimer cases are also conducted. Finally, potential force transmission pathways are discussed based on the dynamics of the glycocalyx constituents, which provides new insight into the mechanism of mechanotransduction of the glycocalyx. These findings have relevance in the pathologies of glycocalyx-related diseases, for example in renal or cardiovascular conditions.

## Keypoints

— Large-scale parallel molecular dynamics simulations are conducted to study the dynamics of flow interacting with endothelial glycocalyx layers.
— For the first time, flow in the physiologically relevant range is realized in a most detailed atomistic model of the glycocalyx.
— The complex dynamics of the glycocalyx relays the essentially one-dimensional forcing imposed to all spatial directions.
— Coupled dynamics exist not only between the flow and the glycocalyx but also within the glycocalyx molecular constituents.
— Shear stress distributes hierarchically inside the glycocalyx layer.
— A potential force transmission pathway is proposed.

## 1. Introduction

The glycocalyx has a prominent role in orchestrating multiple biological processes occurring at the plasma membrane [1]. A glycocalyx layer, a network of membrane-bound proteoglycans and glycoproteins, located on the surface of endothelial cells, is directly exposed to the lumen blood and has complex molecular constituents. The major components of the glycocalyx are glycoproteins bearing acidic oligosaccharides and terminal sialic acids, and proteoglycans with their associated glycosaminoglycan side chains [2]. Experiments have evidenced the glycocalyx's crucial function as a mechanosensor and transducer of fluid shear stress on endothelial cells [2,3], and studies regarding the glycocalyx's role as a mechanotransducer of shear stress [4–7] have prevailed in the realm of glycocalyx-related research. To reveal the mechanotransduction mechanism of the glycocalyx, researchers need a thorough and comprehensive understanding of fluid shear stress variations inside and around the glycocalyx layer. However, it is difficult to gauge the effects of complicated configurations (like the glycocalyx) on flow, let alone precisely describe the variations and distributions of the flow shear stress in this environment. This challenge leaves many fundamental problems unsolved, such as whether shear stress is uniformly distributed in the glycocalyx layer or not, whether different shear stress distributions have distinct effects on the mechanotransduction of the glycocalyx, or how, if at all, the glycocalyx in turn affects the shear stress variations and distributions. These pending issues potentially have a high impact on our understanding of mechanotransduction processes and consequently on pathophysiology.

The difficulty in experimentally obtaining atomic information inside the glycocalyx layer compels researchers to seek insight via computational methods. Current computational studies regarding flow and shear stress in this system are mainly based on continuum and coarse-grained methods. In studies using continuum methods, the flexibility of the soft and easy-to-deform sugar chains is usually neglected or the whole glycocalyx is regarded as rigid [2,4], while, in their coarse-grained counterparts, sugar chains are simplified as polymers with one end tethered on a rigid wall [8]. These simplified studies can partially reveal flow-related phenomena; however, they inevitably neglect the detailed dynamics of the glycocalyx. Thus, fully atomistic molecular dynamics (MD) simulations, including delicate structural information of biomolecular systems, can be a promising, albeit expensive, way to reveal the dynamics of both flow and the glycocalyx. The first (and to our knowledge the only) all-atom glycocalyx system alongside the transmembrane protein, glycosaminoglycans embedded within a lipid bilayer, was recently constructed by Cruz-Chu *et al.* [9]. In order to extract measurable responses from the system, the flow regime was implemented with average velocities of more than 10 m s^−1^. Although quite revealing regarding the dynamics of the system, it is difficult to extract detailed quantitative metrics from a molecular system where the forcing assumes values that are substantially higher than those encountered in real physiology. Therefore, additional studies unravelling the comprehensive dynamics of both flow and the glycocalyx under physiological flow conditions are still needed.

In this paper, an all-atom flow/glycocalyx system will be constructed, and driven with physiologically relevant flow. The dynamics of the flow, including velocity profiles, streamlines and vortices, will be presented. Meanwhile, the diverse behaviour of the glycocalyx amid water molecules will also be outlined. Furthermore, shear stress distributions will be compared between systems with different glycocalyx configurations. We believe that this research sheds light on atomic events inside the endothelial glycocalyx layer, thereby enhancing our understanding of the pathology in glycocalyx-related diseases, such as renal or cardiovascular diseases [10–12].

## 2. *In silico* set-up

### 2.1. The glycocalyx–flow system

The prototype of our simulation system is similar to the one found in Cruz-Chu *et al.*'s [9] equilibrated structure. In the system, syndecan-4 (Syn-4) proteoglycan and heparin sulfate (HS) sugar chains are selected to model the glycocalyx. The glycocalyx structure can be divided into three parts: Syn-4 ectodomain linked with sugar chains; Syn-4 transmembrane dimer embedded into a lipid bilayer; and Syn-4 cytoplasmic dimer.

**Figure 1.**
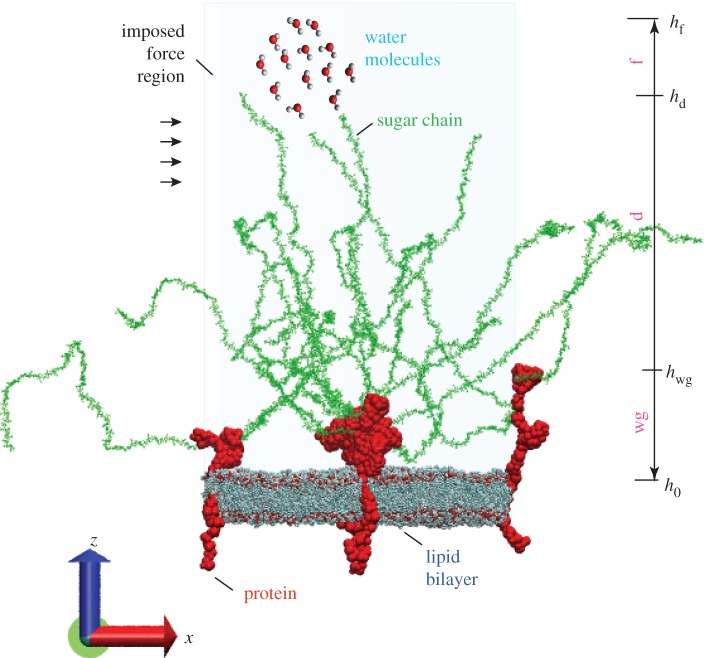
Initial configuration of the all-atom glycocalyx–flow system. The system includes three Syn-4 dimers, 18 sugar chains attached on the apexes of the Syn-4 dimers and a lipid bilayer. External forces are imposed in the *x*-direction on the water molecules in the ectodomain. Water molecules are partially shown, and ions are not shown. (Online version in colour.)

The initial configuration of the glycocalyx–flow system is illustrated in figure 1 and in the electronic supplementary material, movie 1. The whole space is divided into two compartments by the lipid bilayer. Above the lipid bilayer is the ectodomain, which represents the space outside the endothelial cells, where flow passes by. This region contains negatively charged HS sugar chains, the Syn-4 ectodomain in connection with HS sugar chains, water molecules and ions. Below the lipid bilayer is the cytoplasm, which represents the inner space of the cell, which is filled with Syn-4 cytoplasmic protein, water molecules and ions. All the biomolecules are solvated and ionized to 0.1 M NaCl concentration.

In the system, three proteoglycans, which individually consist of one Syn-4 dimer, are embedded in the lipid bilayer. Each proteoglycan has six sugar chains attached on the dimer apexes. To mimic flow, external forces are imposed on the water molecules in the ectodomain. The simulation box is a hexagonal prism with an area of 820 nm^2^ and height of 72 nm. The glycocalyx–flow system comprises approximately 5 800 000 atoms in total.

### 2.2. Protocol details

The TIP3P water model [13] is adopted to simulate water molecules. A CHARMM biomolecular force field [14] has been applied on the proteins and the lipid bilayer. The force field parameters for sugar chains and graphene layers are also adopted from Cruz-Chu *et al*.’s study [9].

We adapted Cruz-Chu *et al.’*s model [9] by rearranging the dimensions of the ectodomain and cytoplasm spaces. Furthermore, to prevent the *z*-direction periodic motion of water molecules from disturbing the microenvironment of the ectodomain and cytoplasm, we also added fixed graphene layers on the top of the ectodomain as Cruz-Chu *et al.* [9] did. (Details about the adaptation can be found in §S.1 of the electronic supplementary material.) We carried out a simulation in an isothermal–isobaric (NPT) ensemble with fixed graphene layers at 1 atm and 310 K using a Langevin thermostat and a Nosé–Hoover Langevin piston for 2 ns, followed by another simulation in a canonical (NVT) ensemble using a Langevin thermostat to maintain the temperature at 310 K for 0.5 ns. The last frame of the NVT simulation was used as the initial configuration (as shown in figure 1) of the follow-up ‘production’ flow simulations. To mimic flow, external forces were imposed on water oxygen in the ectodomain; therefore, the simulation was a non-equilibrium MD simulation. Owing to the high computational expense, the flow simulations were conducted within nanosecond scale. In the flow simulations, a Lowe–Andersen thermostat, a specific thermostat exclusively for flow problems, was selected to maintain the temperature at 310 K.

In the flow simulations, the velocity Verlet integration method [15] was used to advance the positions and velocities of the atoms in time. A 2 fs time step, and particle mesh Ewald electrostatics with a grid density of 1/Å^3^ were used. The SETTLE algorithm [16] was used to enable the rigid bonds connected to all hydrogen atoms. A cut-off of 12 Å with a switching function starting at 10 Å was used to calculate the van der Waals interactions as the previous study [9] did.

All MD simulations were performed using the software NAMD 2.9 [17]. The visualization of the molecular structures was performed with the VMD [18] package. Post-processing of the MD results was accomplished using PYTHON (Python Software Foundation, Wilmington, DE) scripts. All parallel simulations and non-visualized post-processing were conducted on ARCHER, the UK's national supercomputing service. To obtain a simulation result with a physical time of 1 ns, 9000 cores were simultaneously employed for approximately 2 h.

To study the effects of biomolecules on flow, the ectodomain of the system was divided into three sub-regions along the *z*-direction in terms of the different varieties of molecules therein: the near-wall region (wg), the dendritic region (d) and the flow region (f). The heights of the near wall (*h*_wg_ in figure 1), the dendritic (*h*_d_) and the flow sub-regions were 12 nm, 36 nm and 50 nm, respectively. (The details of the division and the determination of the dimensions can be found in §S.4 of the electronic supplementary material.)

## 3. Results

### 3.1. Flow velocity profiles

The flow generated by an external force of 0.003 fN is a laminar flow. (The derivation can be found in §S.5 of the electronic supplementary material.) To understand whether the presence of the glycocalyx could influence the laminar flow, the velocity profiles of the ectodomain water molecules were investigated from both temporal and spatial perspectives.

#### 3.1.1. Spatial distributions of velocities

**Figure 2.**
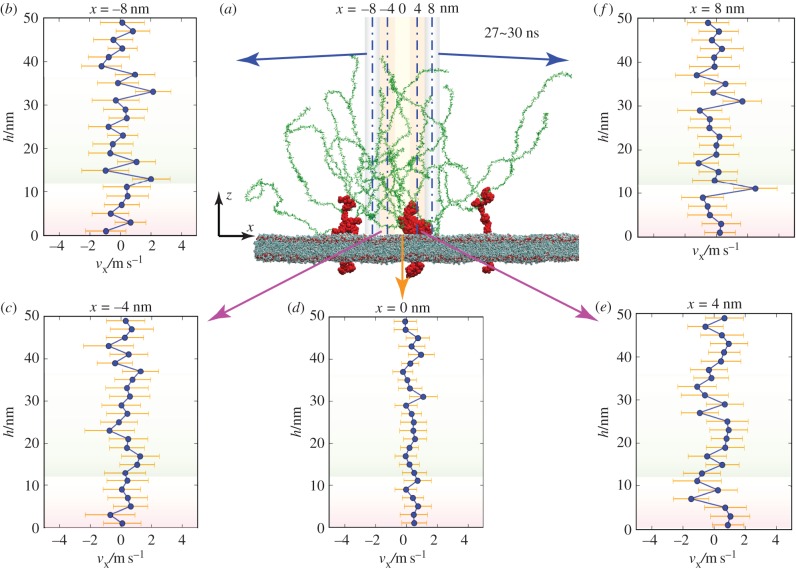
Spatial distributions of velocities from 27 to 30 ns. (*a*) Snapshot of the system configuration at 30 ns without showing the water molecules or ions, and (*b*–*f*) illustrations of the five selected slice positions along the *x*-direction. Time-averaged velocity distributions along the height within five slices with centrelines located at *x* = −8, −4, 0, 4 and 8 nm, respectively. (Online version in colour.)

We first investigated the spatial distribution of the *x*-direction velocities of ectodomain water molecules from 27 to 30 ns. Along the *x*-direction, five slices were extracted in the vicinity of *x* = 0. Each slice had a width of 2 nm in the *x*-direction, and the centrelines of the slices were located at *x*-coordinates equalling −8, −4, 0, 4 and 8 nm, respectively (figure 2*a*). Within each slice, the vertical space was sliced into 25 equal bins along the height (the schematic for the binning method can be found in the electronic supplementary material, figure S4b). For each bin, the *x*-direction velocities of the water oxygens within the slice were averaged. Time-averaging of the velocities from 27 to 30 ns was conducted simultaneously. All five of the selected slices showed zigzag velocity distributions along the height (figure 2*b*–*f*), which differs from the smooth velocity distribution in laminar flow on a bare surface [19]. The difference implies that the presence of the glycocalyx, as an obstacle to the flow, may disturb the fluid flow and alter the velocity distribution. Furthermore, the velocity curves at *x* = 0, where more glycocalyx atoms gather (the glycocalyx atom numbers of the *x* = 0 and *x* = −4 nm regions are 11 368 ± 707 and 6214 ± 404), are smoother than those at *x* = −8 nm (*p* < 0.05) with fewer glycocalyx atoms (with a glycocalyx atom number of 4521 ± 278), which indicates that the glycocalyx molecules may regulate the velocity (*p*-values for the comparisons can be found in the electronic supplementary material, table S3). An additional *in silico* experiment was conducted as the control group to reveal that the zigzag velocity profile mainly comes from the presence of the glycocalyx rather than the noise of the MD method (details can be found in §S.6 in the electronic supplementary material).

#### 3.1.2. Temporal distributions of velocities

**Figure 3.**
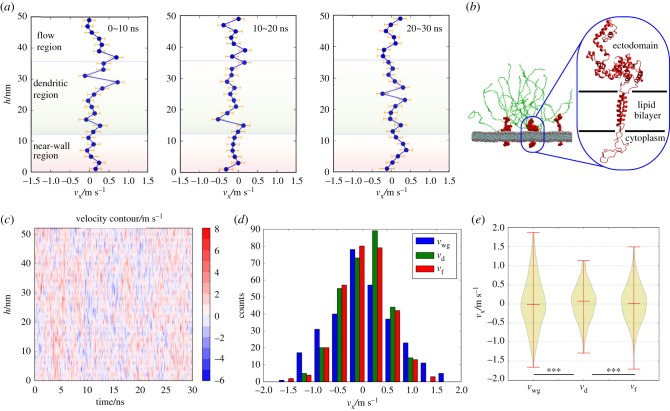
Temporal distributions of velocities at successive intervals and hierarchical velocity distributions across sub-regions. (*a*) Velocity distributions during periods of 0∼10 ns, 10∼20 ns and 20∼30 ns. (*b*) α-Helixes are the main secondary structures of ectodomain proteoglycans. (*c*) Velocity distribution versus height for all recorded frames throughout the 30 ns simulation. (*d*) Distributions of average velocities in each sub-region for all recorded frames throughout the 30 ns simulation. (*e*) Velocities ranges within three sub-regions. The flexible sugar chains can reduce the fluctuations of the flow. Statistical significance is given by: *p < 0.05; **p < 0.01; and ***p < 0.001, and *p*-values can be found in the electronic supplementary material, table S3.

We then studied the evolution of velocity distributions throughout the 30 ns simulation by slicing the whole ectodomain into 25 equal sub-layers (the schematic for a sub-layer can be found in the electronic supplementary material, figure S4a) and averaging the *x*-direction velocities of water molecules in each layer every 10 ns. Compared with the binning method used in studying the velocity spatial distribution, each sub-layer in this section contains more water molecules and more frames are included for averaging. As expected, the zigzag velocity distributions can still be observed at successive instants (figure 3*a*). The zigzag velocity distributions, thus, demonstrated that the glycocalyx atoms continuously disturb water flow and alter the velocity curves throughout the 30 ns simulations.

#### 3.1.3. Hierarchical velocity distributions

In figure 3*a*, hierarchical velocity distributions can also be observed among the sub-regions defined previously. In the near-wall region, although there are some corners, the velocity curve generally tends to be smooth and uniform without significant corners, especially from 10 to 20 ns. By contrast, in the dendritic region corners frequently occur and significant corners can also be observed. The differences in velocity curves can be attributed to the different feature structures of hallmark biomolecules in each sub-region. In the near-wall region, proteoglycans whose secondary structures are mainly composed of α-helixes (figure 3*b*) dominate the flow. Careful examination of the movement of the proteoglycans (which will be discussed in §3.3) shows that the *x*-direction motion of the rigid and stable α-helixes is limited in a confined region due to the lump configuration. By contrast, soft sugar chains in the dendritic region can move flexibly in the space. Thus, the ectodomain proteoglycans obstruct flow mainly by steric hindrance of the lump configuration, while sugar chains disturb the velocity curves by their flexible movement.

To gain additional insight into the hierarchical velocity distributions, we recorded the velocity distributions along the height every 0.1 ns for 30 ns, and depicted velocity contours as shown in figure 3*c*. The alternate distributions of positive and negative *x*-direction velocities both in space and in time once again corroborate that the presence of the glycocalyx significantly disturbs the flow.

For any instant of the 30 ns simulation, we also averaged the instantaneous *x*-direction velocities within the three sub-regions, and then investigated the regional average velocity distributions of all recorded frames. In figure 3*d* and *e*, *v*_wg_, *v*_d_ and *v*_f_ represent the instantaneous *x*-direction in the near-wall, dendritic and flow regions, respectively. The velocity distributions do not significantly differ among these three sub-regions (figure 3*d*, *p*-values between any two distributions are larger than our defined significance level), but a narrow velocity variation in the dendritic region is observed in the violin plot (figure 3*e*). (Tests and *p*-values for figure 3*d* and figure 3*e* can be found in §S.7 in the electronic supplementary material.) The narrow velocity range of the dendritic region in the violin plot implies that the flexible sugar chains can regulate the flow by reducing the flow fluctuations via frequent interactions with water molecules.

### 3.2. Streamlines and vortices

Although external forces are only imposed in the *x*-direction, the complex structures of the biomolecules in space may still extend the influence of the *x*-direction external forces to the other two directions. In the previous section, the velocity distributions along the *z*-direction have been discussed in detail. In the coming section, we will examine the flow profiles involving the *y*-direction.

Streamlines and vortices of the flow have been investigated in three layers parallel to the XOY plane. These three layers are individually extracted from the sub-regions defined previously at heights of 8, 24 and 46 nm, respectively.

#### 3.2.1. Streamline distributions

**Figure 4.**
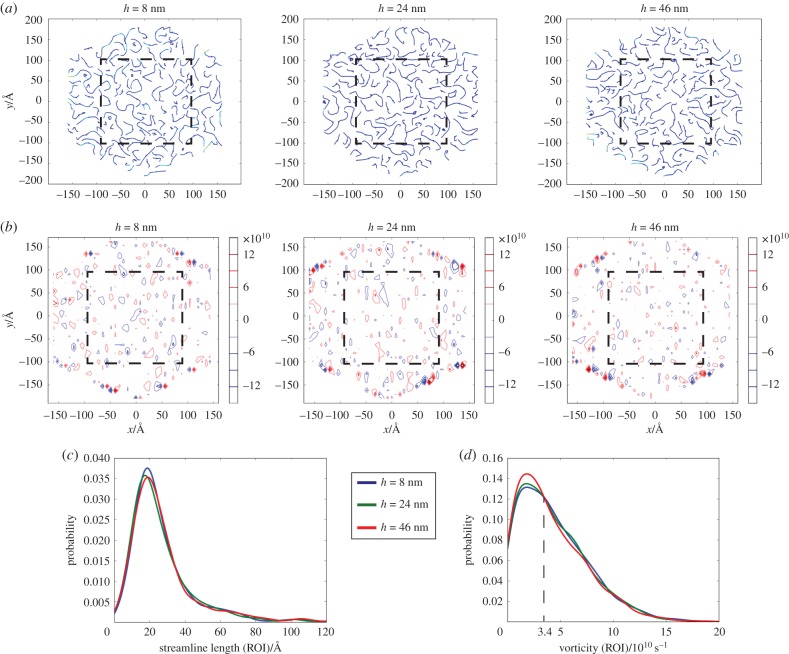
Streamline and vortex distributions of three horizontal layers extracted from three sub-regions. (*a*) Time-averaged snapshot, from 29 to 30 ns, of streamlines in three layers with heights of 8, 24 and 46 nm, respectively. Dash box in each panel marks the region of interest (ROI). (*b*) Time-averaged snapshot, from 29 to 30 ns, of vortices in three layers with heights of 8, 24 and 46 nm, respectively. Dash box in each panel marks the ROI. (*c*) Probability density curves of streamline length for ROIs within three layers. (*d*) Probability density curves of vorticity for ROIs within three layers.

Figure 4*a* shows the average streamline distributions of the three layers from 29 to 30 ns. It is notable that streamlines at the microscale exhibit non-continuous distributions. To further distinguish the streamlines, the probability density functions (PDFs) of the streamline lengths in the region of interest (ROI) were investigated (figure 4*c*). (The convergence of the probability density curves can be found in §S.8 in the electronic supplementary information.) However, neither figure 4*c* nor electronic supplementary material, figure S6a shows significant differences in the probability density distributions of the streamline lengths among these three sub-regions. Thus, we suspect that the glycocalyx constituents may not exert any effects on the streamline lengths.

#### 3.2.2. Vortex distributions

Vortices of the three layers are also depicted (figure 4*b*), and the vorticity (i.e. strength of the vortices) distributions have been investigated (figure 4*d*). As shown in figure 4*d* and electronic supplementary material, figure S6b, when vorticity is weaker than 3.4 × 10^10^ s^–1^, vortices in the flow region have the highest probability density. In other words, the glycocalyx favours strong vortices. The enhanced vorticity by the glycocalyx may be attributed to the swirling motions of both proteins and sugar chains (as will be shown in the coming section) which can generate local eddies. Meanwhile, between the near-wall and dendritic regions, a slightly higher probability of vorticities stronger than 3.4 × 10^10^ s^−1^ can be observed in the former, which may be due to the more compact structures of protein in the near-wall region, compared with the loose layout of sugar chains in the dendritic region, as local vortices tend to be generated when flow passes through blunt bodies [20,21].

### 3.3. Dynamics of the glycocalyx

As mentioned previously, the complexity of the glycocalyx structures extends the influence of the *x*-direction external forces to the other two dimensions. To gain additional insight into the coupled dynamics between flow and the glycocalyx, and also for the sake of simplification, we further scrutinize the motion of the central glycocalyx element.

**Figure 5.**
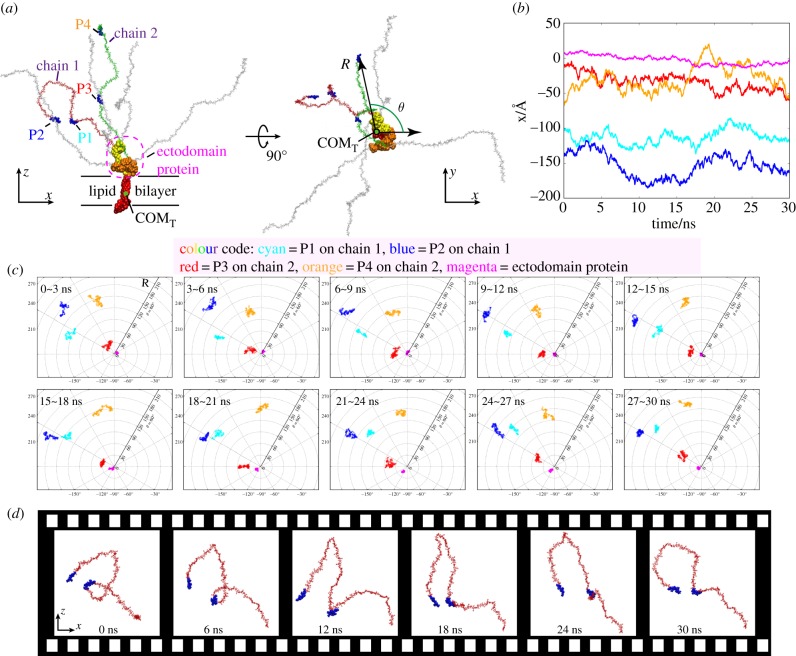
Dynamics of the glycocalyx. (*a*) Five segments of interest and definition of the polar coordinates. The snapshot is taken at the instant of 30 ns. In the following analyses, the positions of COM_T_ in all the frames in the 30 ns simulations are aligned. (*b*) The selected five segments swing in the *x*-direction. (*c*) Evolutions of the COMs of the five segments of interest in polar coordinates. The colour code is valid for (*b*) and (*c*). (*d*) Successive snapshots of the sugar chain undoing its coil via wavy movement.

Five segments, including ectodomain protein and four residue segments on two typical sugar chains, have been selected for motion inspection, as illustrated in figure 5*a*. Segments P1 and P2, individually composed of five residues, are located at 40% of the total length of chains 1 and 2, respectively. Segment P3 and segment P4 each comprise the five ending residues of sugar chain tails. In the following analyses, the positions of the centre of mass of the transmembrane protein (COM_T_) will be aligned for all the frames of the 30 ns simulations, and polar coordinates will also be employed (figure 5*a*). In the polar coordinates system used, *R* refers to the projection of the distance between the COM_T_ and the centre of mass (COM) of the residue of interest on the XOY plane, and *θ* represents the angle between *R* and the +*x*-direction.

The fluctuations of the positions in the *x*-direction (figure 5*b*) reveal the swing motions of the five selected segments. Specifically, segments on the sugar chains (P1–P4) fluctuate more fiercely than the ectodomain protein, which corroborates our previous assertion (in §3.1.3) that the soft and flexible sugar chains exert a stronger disturbance on the water flow than the ectodomain protein does. Meanwhile, the trajectories of the COM of each segment have been recorded in polar coordinates (figure 5*c* and electronic supplementary material, movie 2). The 30 ns simulation witnesses frequent swirling motions of the five selected segments. The 10 snapshots in figure 5*c* also exhibit varying relative positions of the five segments, which depict the unsynchronized motion of the sugar chains and the ectodomain core protein, thereby illustrating a weak correlation of the motions of these components.

In addition, interesting phenomena were also observed amid the complicated motion of the sugar chains. For example, at the start of the simulation, chain 1 exhibits a coiled state from the side view (figure 5*d*). As flow passes, a wave generated by the swing of the linking ectodomain proteoglycan travels along chain 1, and the sugar chain gradually uncoils via this wavy movement (electronic supplementary material, movie 3). This interesting behaviour of the sugar chain further confirms that coupled dynamics prevails within the glycocalyx constituents. The uncoiling process of the sugar chain also conveys its biological significance: the corresponding conformational changes may contribute to an increase in Na^+^ binding sites that initiate signal transduction pathways [22].

### 3.4. Shear stress

As mentioned in the introductory section, to study the mechanotransduction of the glycocalyx under flow shear stress, the shear stress distribution inside and around the glycocalyx layer should be first clarified. Thus, we also studied the flow shear stress in the ectodomain. The focus of our attention in this section is one component of the stress tensor—the shear stress on the plane normal to the *x*-axis in the *z*-direction, *τ*, which is calculated by3.1
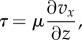
where *μ* is the viscosity of the TIP3P water model (0.321 mPa s) [23] and ∂*v_x_*/∂*z* is the gradient of the *x*-velocity along the *z*-axis.

**Figure 6.**
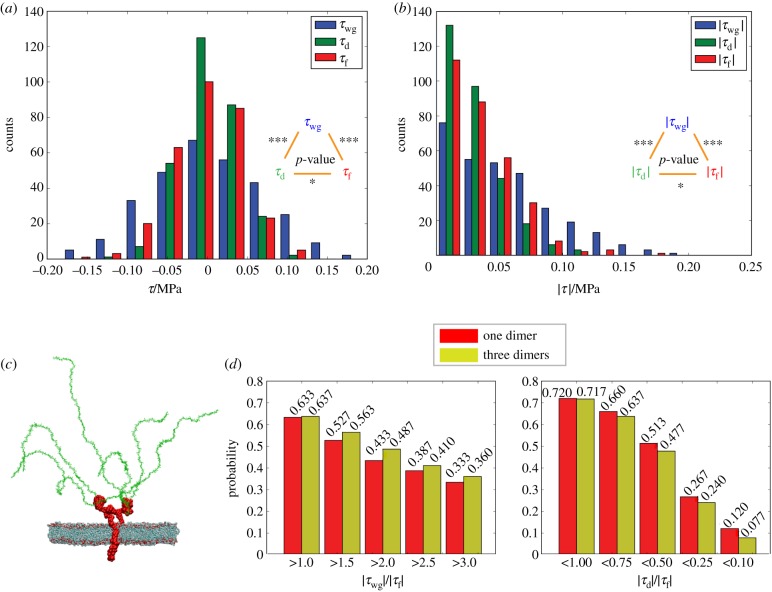
Shear stress distributions in the three sub-regions. (*a*) Shear stress distributions with the *p*-values of K–S tests for each of two distributions. (*b*) Distributions of shear stress magnitudes with the *p*-values of K–S tests for each of two distributions. (*c*) Configuration of the one-dimer *in silico* experimental system as the control group. Water and ions are not shown. (*d*) Comparisons of probabilities to obtain strong shear stress in the near-wall region and to obtain weak shear stress in the dendritic region between the three-dimer and the one-dimer cases. Statistical significance is given by *p < 0.05; **p < 0.01; ***p < 0.001; values are listed in the electronic supplementary material, table S3.

The forward difference method is used to compute the shear stress of interest. (Details about the calculation of the shear stress can be found in §S.9 of the electronic supplementary material.) For every 0.1 ns of the 30 ns simulation, instantaneous shear stress has been averaged within each of the three sub-regions. The count distributions of the shear stresses of the three sub-regions are depicted, and Kolmogorov–Smirnov (K–S) tests have demonstrated that shear stresses in the three sub-regions follow hierarchical distributions as shown in figure 6*a*. Furthermore, the shear stress magnitude distributions in the sub-regions are studied (figure 6*b*), as the magnitudes are related to the deformations of biomolecules. The magnitude distributions reveal that the dendritic region has the weakest shear stress, as the majority of the shear stresses within the region have relatively small magnitudes; by contrast, shear stresses in the near-wall region are the strongest, followed by their flow-region counterparts. The magnitudes of the shear stresses, thus, follow the order: |*τ*_wg_| > |*τ*_f_| > |*τ*_d_| (|*τ*_wg_| > |*τ*_f_| with *p*-value = 2.5 × 10^−9^ < 0.001, and |*τ*_f_| > |*τ*_d_| with *p*-value = 0.0195 < 0.05).

The shear stress distributions can also be attributed to the complicated configuration of the glycocalyx. In the dendritic region, the sugar chains dissipate the momentum of the fluid via frequently interacting with the water molecules as well as disturbing the motions of water molecules, thereby reducing the gradient of the *x*-velocity along the *z*-axis (already discussed in figure 3*e*). While, in the near-wall region, the intense interactions between the lipid bilayer and the adjacent water molecules will act through a friction-like effect to the flow and result in an obvious gradient of the *x*-velocity along the *z*-axis within the layer, beyond the disturbance the ectodomain proteins may introduce to the velocity profile in the *z*-direction.

An additional *in silico* experiment with only one dimer and six sugar chains amid water molecules, ions and the lipid bilayer is included for comparison (figure 6*c*). External forces of 0.003 fN are imposed on the ectodomain water molecules, and the simulation lasts for 15 ns. The probabilities of obtaining |*τ*_wg_| > |*τ*_f_| in both one-dimer and three-dimer cases are calculated. The probability of enhanced near-wall shear stress in the three-dimer case is higher than in its one-dimer case counterpart (|*τ*_wg_|/|*τ*_f_| > 1 in figure 6*d*), especially for extra strong shear stress (|*τ*_wg_|/|*τ*_f_| > 1.5, 2, 2.5 and 3 in figure 6*d*). Thus, it can be inferred that the structure lump of the core proteins in the near-wall region primes the generation of strong shear stress, especially for the generation of extra strong shear stress.

In the dendritic region, shear stress is weakened in both the one-dimer and the three-dimer cases. However, weak shear stress (|*τ*_d_|/|*τ*_f_| < 1 in figure 6*d*), especially extra weak shear stress (|*τ*_d_|/|*τ*_f_| < 0.75, 0.5, 0.25 and 0.1 in figure 6*d*), is less frequently observed in the three-dimer case than in the one-dimer case, which can be due to the excessive interactions of sugar chains and the flow in the three-dimer case. The excessive interactions disturb the flow velocity profile, albeit regulating velocities.

## 4. Discussion

The determination of velocity and force ranges will be discussed first. Cruz-Chu *et al.* [9] mimicked flow passing through an endothelial glycocalyx layer (EGL) by applying external forces of 0.001 pN on every oxygen atom of water molecules on the ectodomain side. The resulting average velocity of the bulk flow under this external force can be as large as 10 m s^−1^, which is substantially higher than previous experimental results [24]. In fact, the order of the velocities for EGLs is expected to be 0.001 ∼ 1 mm s^–1^. (Detailed analysis about the order of the velocities can be found in §S.2 of the electronic supplementary material.) To generate a physiological flow, we systematically decreased the external force on every oxygen atom of the water molecules in the ectodomain. After iterations, we finally selected 0.003 fN as the external force, and prolonged the flow simulation times to 30 ns (further discussion can be found in §S.2 of the electronic supplementary material).

The dynamics of the glycocalyx constituents and mechanotransduction will also be discussed. One key issue in understanding the mechanism of the mechanotransduction of the glycocalyx is to sort out the route via which the flow shear stress is transmitted into the cytoplasm. Previous studies [4,25] favour that the flow shear stress is transmitted via the ‘flow–sugar chains–core protein–cytoskeleton’ route. In our research, by scrutinizing the motions of the sugar chains and the core protein, we suggest that forces can also be transmitted directly from the flow to the core protein, without the transduction of the sugar chains, due to the weak correlation of the motions between the sugar chains and the core protein. These two routes are both potential force transmission pathways via the glycocalyx. Follow-on studies are being undertaken to reveal the transmission mode via the transmembrane core protein to the cytoskeleton.

Molecular dynamics methods used in this research provide the possibility to understand some concepts involved in continuum studies from the atomic perspective. For example, in a previous continuum study [25], the authors discussed the bending rigidity of the core proteins that enables them to resist the deformation by fluid shear stress. Indeed, as illustrated in the secondary structure of the core protein (figure 3*b*), the ectodomain and the transmembrane parts of the core protein are connected by single coils, which also implies unsynchronized motion of the two parts. The presumed deformation by fluid shear stress can be attributed to the unsynchronized motion of the two subdomains of the core protein, with the bending rigidity being a measure of this lack of synchronization.

This research is based on a simple idealized scenario of the endothelial glycocalyx layer influenced by water molecules and ions, without any plasma proteins included. In reality, the composition and thickness of the membrane-associated glycocalyx layer are very dynamic, and are continuously affected by the dynamic equilibrium between the soluble components (such as plasma protein) and other blood constituents [26]. The intricate mesh of the glycocalyx amid various plasma polysaccharides has not been considered in this model.

## 5. Concluding remarks

We use large-scale molecular dynamics simulation to study the dynamics of flow interacting with endothelial glycocalyx layers. For the first time, flow in the physiologically relevant range is realized in a most detailed atomistic model of the glycocalyx. The coupled dynamics of flow and glycocalyx behaviours are presented and analysed. The one-dimensional external force imposed on ectodomain water molecules first drives the flow to move, invoking a complex dynamic response of the glycocalyx. With its complex configurations, the glycocalyx exhibits three-dimensional motions such as swing and swirling, spreading the influence of the external forcing in one direction to the other two directions. Consequently, the glycocalyx disturbs the flow by altering the velocity profiles and modifying the vorticity distributions. Coupled dynamics also exist within the glycocalyx molecular constituents, including sugar chains and proteoglycan. Finally, comparisons of shear stress distributions between the one-dimer and three-dimer cases are conducted. Also, potential force transmission pathways are discussed. New insight gained into the mechanism of mechanotransduction of the glycocalyx in this study has implications for control of glycocalyx-related diseases, for example in renal or cardiovascular conditions.

## Supplementary Material

Simulation details, Statistical Information, and Detailed and Supplementary post-processing and discussion

## Data accessibility

All data supporting this study are provided as electronic supplementary information accompanying this paper.
