# Supplementary material for: Large-scale molecular dynamics simulation of coupled dynamics of flow and glycocalyx: towards understanding atomic events on an endothelial cell surface
Source: J R Soc Interface. 2017 Dec 6;14(137):20170780. doi: 10.1098/rsif.2017.0780 (PMC5746579; doi:10.1098/rsif.2017.0780)
Supplement: Simulation details, Statistical Information, and Detailed and Supplementary post-processing and discussion [file rsif20170780supp1.pdf]

Supporting Information

for

# Large-Scale Molecular Dynamics Simulation of Coupled Dynamics of Flow and Glycocalyx: Towards Understanding Atomic Events on Endothelial Cell Surface

Xi Zhuo Jiang[1], Haipeng Gong[2], Kai Hong Luo[1*], Yiannis Ventikos[1*],

1. Department of Mechanical Engineering, University College London, Torrington Place, London WC1E 7JE, UK
2. MOE Key Laboratory of Bioinformatics, School of Life Sciences, Tsinghua University, Beijing, 100084, P. R. China

***** Email address for correspondence:

k.luo@ucl.ac.uk (Kai Hong Luo)

y.ventikos@ucl.ac.uk (Yiannis Ventikos)

## S.1 Simulation details

To mimic the vascular flow, we impose *x*-direction external forces on oxygen atoms of water molecules in the ectodomain. Since periodic boundary conditions are applied on three directions, there would be some water molecules with imposed force in the ectodomain propagating to the cytoplasmic side through the periodic boundary in the *z* direction. To prevent the propagating water molecules from disturbing the micro-environment of the cytoplasm, graphene layers with total thickness of 2 nm, located 58 nm above the lipid membrane, have been added on the top of the system **Figure S 1**), forming an effective division between the ectodomain and the cytoplasm. Thus, the space along the *z* direction has been divided into two compartments: the ectodomain compartment, containing all HS sugar chains, Syn-4 ectodomain in connection with HS sugar chains, water molecules and ions, which starts from the lipid membrane top surface to the base of graphene layers; and the cytoplasmic compartment, with Syn-4 cytoplasmic subdomain inside, which occupies the space beneath the membrane.

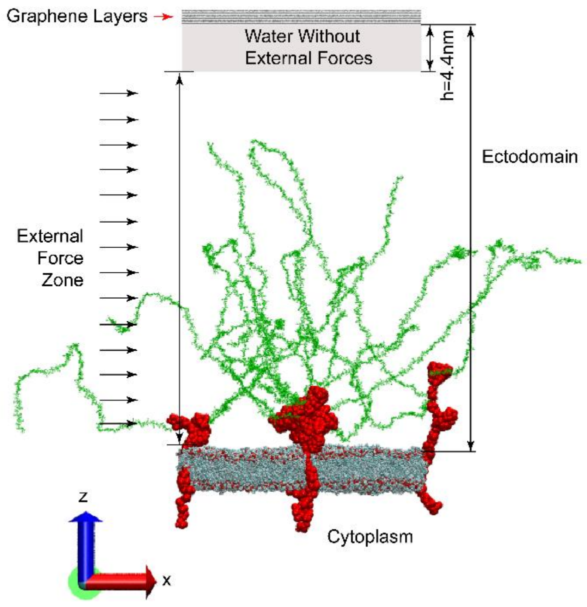


**Figure S 1 Schematic of the flow/glycocalyx system with graphene layers. Graphene layers are added on the top to prevent the water molecules propagating from ectodomain to the cytoplasm due to the periodic boundary conditions.**

As mentioned in the main text, our glycocalyx system is adapted from Cruz-Chu's [1] equilibrated structure. In their structure, no graphene layers are provided, and the ectodomain and cytoplasm are of equal dimensions. Since we focus on the flow in ectodomain and graphene layers are required as boundary, we first rearrange the water distribution by moving the basal cytoplasmic equilibrated water molecules to the ectodomain apical region, followed by graphene layers being added on the top of the newly formed ectodomain. A simulation using an NPT ensemble with graphene layers being fixed is conducted at 1 atm and 310K using a Langevin thermostat and a Nosé-Hoover Langevin piston for 2 ns, followed by another simulation in a NVT ensemble using a Langevin thermostat to keep temperature at 310K for 0.5 ns. The last frame of the NVT simulation, then, is used as the initial configuration (as shown in Figure 1 in the main text) of the follow-up flow simulations. In the flow simulations, the Lowe-Andersen thermostat has been selected to maintain temperature at 310K.

## S.2 External force and resulting flow

Cruz-Chu [1] mimicked flow passing through an endothelial glycocalyx layer (EGL) by applying external forces of 0.001pN on every oxygen atom of water molecule in the ectodomain side. The resulting average velocity of the bulk flow under this external force can be as large as 10 m/s, which is higher compared with previous experimental results [2]. To get an overview of the order of the velocity in the endothelial glycocalyx layer, we conducted the following derivation.

The thickness of an endothelial glycocalyx layer is usually less than 500 nm, and in Cruz-Chu's [1] and our cases the EGL is 50 nm high. Poiseuille's law is used to estimate the order of magnitude of the velocity for the EGL, and schematic for estimating the velocity is shown in **Figure S 1**. For a pipe flow, the velocity ($u$) at a certain radius and the maximum velocity ($u_{\max}$) follows

$$u_{\max} = u\left(\frac{R^2 - r^2}{R^2}\right) \qquad \text{(S1)},$$

where *R* is the radius of the vessel, and *r* is the distance between the top of the EGL and the centre of the vessel.

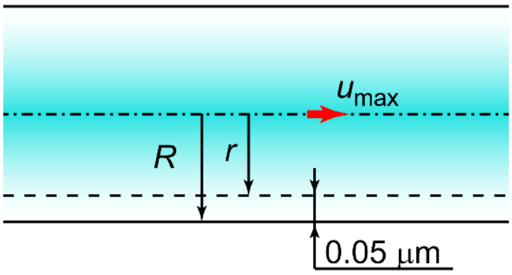


**Figure S 2  Schematic for estimating the order of magnitude of velocities in the glycocalyx layer.**

Capillary and aorta are taken as instances here. Estimated orders of magnitude of peak velocities of the EGL for both types of vessels are summarised in **Table S 1**.

**Table S 1  Estimated orders of magnitude of peak velocities of EGL**

| Vessel Type | $R$ | $u_{max}$ | $u$ / mm·s$^{-1}$ |
|---|---|---|---|
| capillary | 1~10 μm [3] | ~ cm/s [2] | 0.1~1 |
| aorta | ~ cm [4] | 0.1 ~ 1 m/s [5, 6] | 0.001~0.01 |

Thus, the order of the velocities for EGLs is expected to be 0.001 ~ 1 mm/s.

To generate a physiological flow, we systematically decreased the external force on every oxygen atom of water molecules in the ectodomain, and then calculated the average *x*-direction velocity of the bulk flow ($v_x$) by averaging all the *x*-direction velocities of water oxygen atoms in the ectodomain. Through iterations, we found that with external force of 0.002 fN~0.0035 fN, the average of $v_x$ fluctuates in the vicinity of 0 m/s. This is consistent with the Weinbaum *et al* [7] estimation. Considering the computational expense in covering all the velocities in the range of 0.001 to 1 mm/s, the current velocity range under the external force of 0.003fN is chosen. Thereafter, we selected

0.003fN as external force, and prolonged the flow simulation to 30 ns. **Figure S 3**a illustrates the *x*-direction velocity time evolution at external force of 0.003fN. To reduce noise in the simulations, time-averaging of the velocities at different intervals has also been conducted.

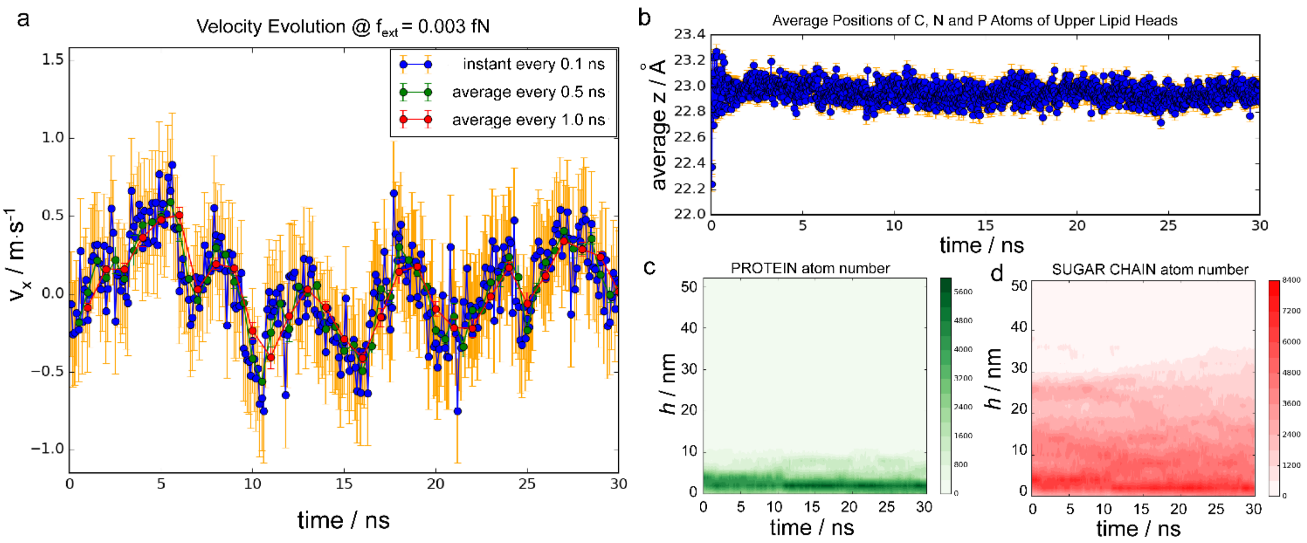


**Figure S 3 Velocity evolution of water molecules and geometric information of the glycocalyx-flow system.** a. Evolution of x-direction average velocity of oxygen atoms of water molecules in the ectodomain under the external force of 0.003fN. Blue dots represent the instantaneous average velocity at every 0.1 ns; Green and red dots represent the time-averaged velocity with intervals of 0.5 ns and 1 ns, respectively. b. Average positions of C, N and P atoms of upper lipid heads during the 30-ns simulation. c. Atom number distribution of proteins above the lipid membrane throughout the 30-ns simulation. d. Atom number distribution of sugar chains throughout the 30-ns simulation.

## S.3 Binning methods in this research

In this research, to study flow structures, different binning methods were used. **Figure S 4** illustrates binning methods used in this research.

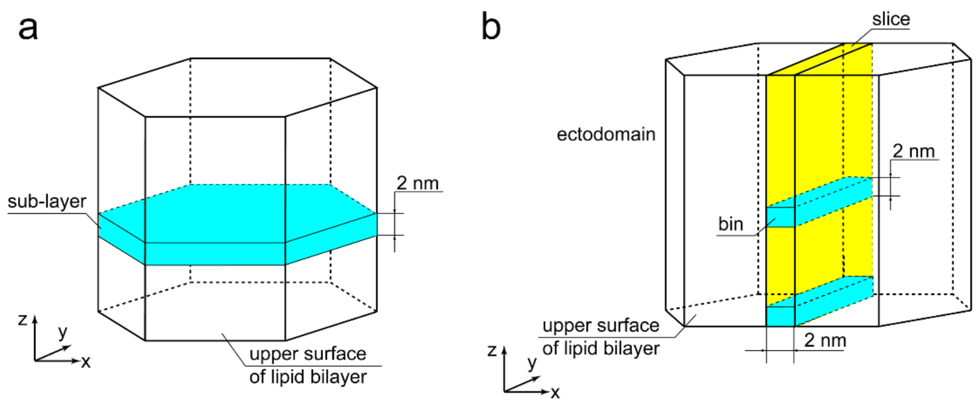


**Figure S 4  Schematic of binning methods used in this research**

## S.4 Details for geometric division

As mentioned in the main text, the ectodomain can be categorised into three sub-regions in terms of molecule varieties therein. The near wall region, starting from the upper surface of the lipid bilayer and ending at the apex of Syn-4, contains Syn-4 ectodomain, a few sugar chains, water molecules and ions. The dendritic region, from the apex of Syn-4 to the furthest end of the sugar chains and containing sugar chains, water molecules and ions, features its tree-like structure.The flow region, above the dendritic region, only includes water molecules and ions. To measure the dimension of each sub-region, we first determined the height origin of the ectodomain space ($h_0$ in Figure 1 in the main text) by the average positions of heavy atoms of the upper lipid heads.

To draw the boundaries of the sub-regions, the height origin of the ectodomain ($h_0$ in Figure 1 of the main text) needs to be first determined. The average position of heavy atoms (including carbon, nitrogen and phosphor) of the upper lipid heads throughout the 30-ns simulation is appointed as the height origin. During the 30-ns simulation, the average positions of the heavy atoms fluctuate at 23 Å (23 Å is the absolute position in the z direction) as shown in **Figure S 3**b, which will be used as the height origin of the ectodomain in further discussion.

The dimension of each sub-region is then determined by the steric information of hallmark biomolecules during the 30-ns simulation. Apexes of proteoglycans and sugar chains are individually declared as boundaries of near wall and dendritic sub-regions. To locate the apexes, the ectodomain

originating from the height origin (z coordinate equalling 23 Å) to 50 nm above has been equally sliced into 25 sub-layers with layer thickness of 2 nm (Figure S4a). Within each sub-layer, the numbers of proteoglycan and sugar residue atoms have been counted. The apical positions of the character biomolecules are then determined by the highest layers containing the biomolecules of interest among all frames of the 30-ns simulation. **Figure S 3c** and **d** illustrate the atom number distributions of proteoglycan and sugar residue atoms throughout the 30-ns simulation. According to the distributions, the apexes of proteoglycans and sugar chains are located at 12 nm and 36 nm, respectively. Thus, the heights of the near wall sub-region ($h_{wg}$ in Figure 1 of the main text) and the dendritic sub-region ($h_d$ in Figure 1 of the main text) are set to 12 nm and 36 nm, together with $h_f$ equalling 50 nm as designated.

## S.5 Judgement for laminar flow

Reynolds number, Re, is defined as

$$\mathrm{Re} = \frac{\rho u l}{\mu} \qquad \text{(S2)},$$

where $\rho$ is the fluid density, $u$ is the flow velocity, $l$ is the characteristic length and $\mu$ is viscosity. In this research, water density is $10^3$ kg/m$^3$, the characteristic length is 50 nm, and viscosity of TIP3P water model is 0.321 mPa·s [8]. According to the velocity evolution in Figure S3a, the maximum flow velocity is smaller than 1 m/s under the external force of 0.003fN. Incorporating these values then gives the estimation of Re to be far less than 100 (<< 10,000), so the flow is classified as laminar flow.

## S.6 *In Silico* experiment as control group

To verify whether the zigzag velocity profiles (Figures 2 and 3 in the main text) stem from the presence of the glycocalyx or they are consequences of noise of the MD methods, we conducted an additional *in silico* experiment as control. In this experiment, an equilibrated system with only water molecules, ions and the lipid membrane was selected as initial configuration (**Figure S 5**). All the water molecules are located above the lipid membrane. The dimension of the system is 135 × 140 ×

375 Å$^3$ with the height of water being 330 Å. The whole system comprises about 391,000 atoms. We conducted equilibrium simulations for 1 ns in the NVT ensemble with the Lowe-Andersen thermostat as used in the flow simulation.

In the post-processing of the flow simulation, when the ectodomain is divided into 25 sub-layers (as shown in **Figure S 4**a), each layer contains 40,000 to 50,000 water molecules. To be consistent with the flow simulation, we divided the water region of the control group into two layers each of which contains 38,000 to 43,000 water molecules. We then calculated the average *x*-direction velocities of both layers for each frame, followed by calculating the average velocity differences between the layers every 5 frames. So we obtained a distribution of the velocity differences of the control group.

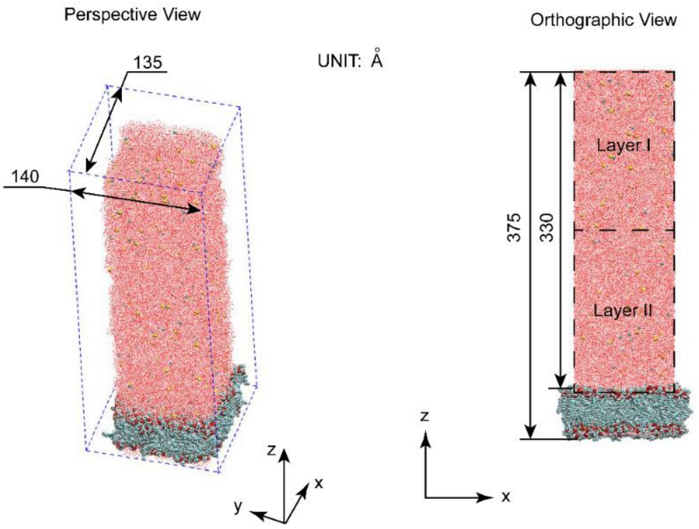


**Figure S 5 Initial configuration of the control group and division of the water box.**

Similarly, we calculated the velocity differences between two adjacent sub-layers in each sub-region of the flow system in the main text, and averaged the velocity differences every 5 frames. Also, we obtained three distributions of the velocity differences for the three sub-regions.

Finally, we compare the distributions of velocity differences between the control group and every sub-region using a Kolmogorov-Smirnov test; the P-values are listed in **Table S 2**. The null hypothesis

of the Kolmogorov-Smirnov test is that the two samples are drawn from the same distribution, so we can conclude that the zigzag velocity profile is not from MD noise, but mainly stems from the disturbance of the glycocalyx constituents.

**Table S 2 P-values of Kolmogorov-Smirnov tests for the control group and the flow simulation**

| Samples | P-values |
|---|---|
| (near wall, control) | 0.004 |
| (flow, control) | 0.007 |
| (dendritc, control) | 0.004 |

## S.7 Statistical tests in this research

**Table S 3 P-values of tests**

| Figure in the main text | Samples | Tests | P-values |
|---|---|---|---|
| Figure 2 | Velocity var($x$ = 0, $x$ = -8 nm) | Bartlett | 0.038 |
| | Velocity var($x$ = -4 nm, $x$ = -8 nm) | | 0.030 |
| Figure 3d | ($v_{wg}$, $v_d$) | Kolmogorov-Smirnov | 0.06 |
| | ($v_d$, $v_f$) | | 0.84 |
| | ($v_{wg}$, $v_f$) | | 0.14 |
| Figure 3e | Var($v_{wg}$, $v_d$) | Fligner | $1\times10^{-6}$ |
| | Var($v_d$, $v_f$) | | $2.9\times10^{-5}$ |
| | Var($v_{wg}$, $v_f$) | | 0.28 |
| Figure 6a | ($\tau_{wg}$, $\tau_d$) | Kolmogorov-Smirnov | $7\times10^{-5}$ |
| | ($\tau_d$, $\tau_f$) | | 0.049 |
| | ($\tau_{wg}$, $\tau_f$) | | $7\times10^{-5}$ |
| Figure 6b | ($\lvert\tau_{wg}\rvert$, $\lvert\tau_d\rvert$) | Kolmogorov-Smirnov | $2\times10^{-16}$ |
| | ($\lvert\tau_d\rvert$, $\lvert\tau_f\rvert$) | | 0.039 |
| | ($\lvert\tau_{wg}\rvert$, $\lvert\tau_f\rvert$) | | $5\times10^{-9}$ |
| Figure 6d | one-dimer ($\lvert\tau_{wg}\rvert$, $\lvert\tau_f\rvert$) | Kolmogorov-Smirnov | $2\times10^{-5}$ |
| | one-dimer ($\lvert\tau_d\rvert$, $\lvert\tau_f\rvert$) | | 0.13 |
| | one-dimer ($\lvert\tau_{wg}\rvert$, $\lvert\tau_d\rvert$) | | $8\times10^{-9}$ |
| | $\lvert\tau_{wg}\rvert/\lvert\tau_f\rvert$ (one-dimer,three-dimer) | Chi-square | 0.61 |
| | $\lvert\tau_d\rvert/\lvert\tau_f\rvert$ (one-dimer,three-dimer) | | 0.71 |

## S.8 Distributions of streamline lengths and vorticity

Streamlines and vortices of the flow have been investigated in three layers parallel to the XOY plane. These three layers are individually extracted from the near-wall, dendritic and flow sub-regions at heights of 8 nm, 24 nm and 46 nm, respectively.

The streamline and vortex distributions in Figure 4a and Figure4b are time-averaged results for 5 instants, including 29.2, 29.4, 29.6, 29.8 and 30 ns. To find the distribution differences among the three layers, the probability density curves for the three layers are compared. Before comparisons, the probability density curved were first validated convergent by checking whether probability density curves change significantly when more data is included. As shown in **Table S 4**, when time increases to 30 ns, the probability density curves for streamlines and vorticity in three layers remain stable. Thus, the probability density curves for streamline lengths and voriticity are convergent, and in the main text we use the probability density curves enumerating from 29.2 to 30.0 ns to compare streamlines and vorticity in the three layers of interest.

## Table S 4 Convergence of the Streamline Length and Vorticity Probability Density Distribution

<table>
<tr><th></th><th>h = 8 nm</th><th>h = 24 nm</th><th>h = 46 nm</th></tr>
<tr><td>Streamline length</td><td>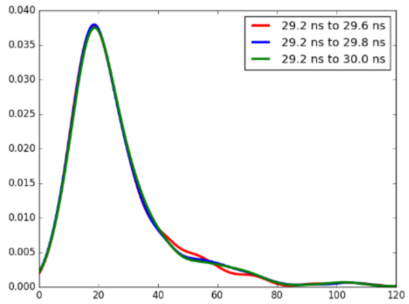</td><td>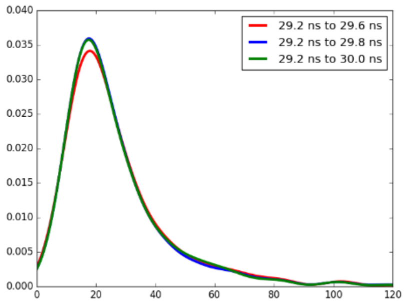</td><td>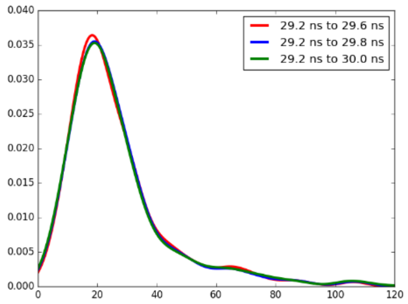</td></tr>
<tr><td>Vorticity</td><td>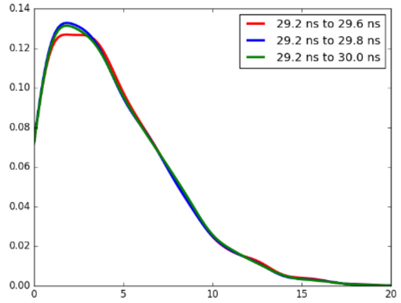</td><td>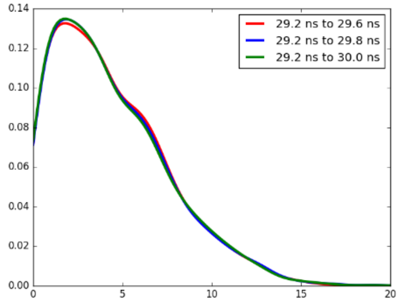</td><td>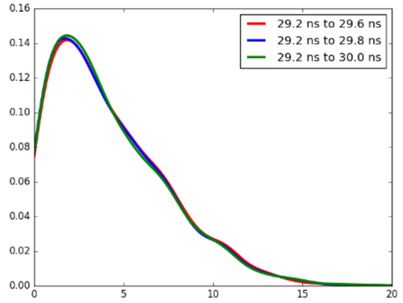</td></tr>
</table>

Cumulative density functions (CDFs) of the streamline length and vorticity of the three selected layers are also calculated, as shown in **Figure S 6**. The three CDF curves of streamline length in **Figure S 6**a are entangled, which implies that the glycocalyx constituents may not exert effects on the streamline lengths. By contrast, in **Figure S 6**b, when vorticity is weaker than $3.4 \times 10^{10}$ $s^{-1}$, vortices in the flow region (h=46 nm) have the highest probability density, which means that the glycocalyx favours strong vortices. Between the near-wall and dendritic regions, a slightly higher probability of vorticities stronger than $3.4\times10^{10}$ $s^{-1}$can be observed in the former (h=8 nm).

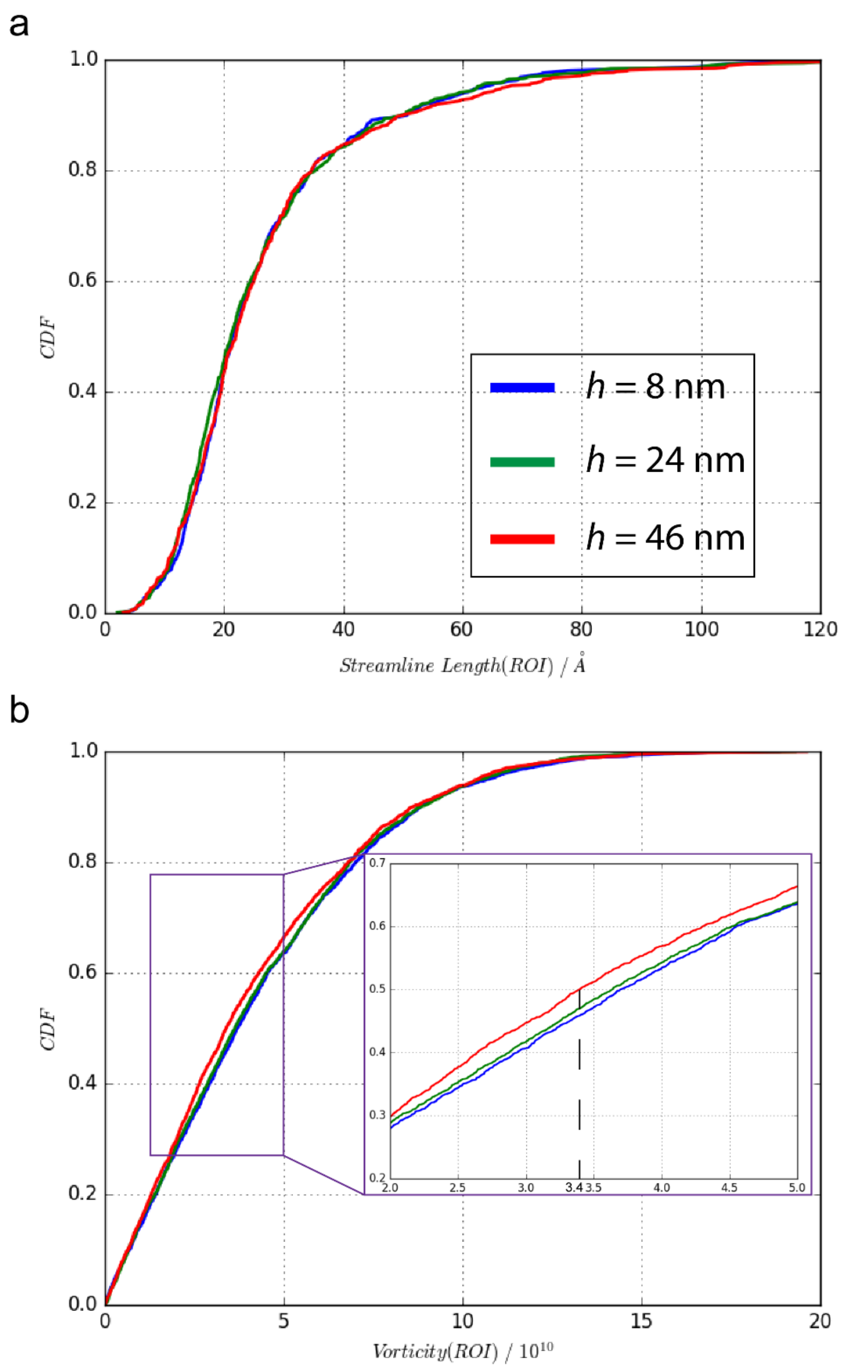


**Figure S 6 CDFs of streamline lengths and vorticity of the three selected layers.** (a) CDFs of streamline lengths; and (b) CDFs of vorticity. Legend is valid for both panels.

## S.9 Calculation of the shear stress

Discrete form of Eq. (1) in the main text can be written as Eq. (S3) by forward difference method.

$$\tau_i = \mu \frac{v_{x,i+1} - v_{x,i}}{z_{i+1} - z_i} \tag{S3}$$

In Eq.(S3), index $i$ ($0 < i < 25$) represents the $i$-th sub-layer defined in Figure S2a, and the denominator, indeed, is the height of each sub-layer (2 nm).

The shear stress in each sub-region ($\tau_{\mathrm{wg}}$, $\tau_{\mathrm{d}}$, and $\tau_{\mathrm{f}}$) is then calculated by averaging sub-layer shear stresses therein. For example, the near-wall region is 12 nm in height and contains 6 sub-layers. For a certain instant, the average shear stress in the near-wall region, $\tau_{\mathrm{wg}}$, is calculated as

$$\tau_{wg} = \frac{1}{5}\sum_{i=1}^{5} \tau_i \tag{S4}$$

Analogously, average shear stresses in the dendritic and flow sub-regions are

$$\tau_d = \frac{1}{11}\sum_{i=7}^{17} \tau_i \tag{S5}$$

$$\tau_f = \frac{1}{6}\sum_{i=19}^{24} \tau_i \tag{S6}$$

The magnitudes of the three sub-regional shear stresses, for a certain instant, are

$$\left|\tau_{wg}\right| = \left|\frac{1}{5}\sum_{i=1}^{5} \tau_i\right| \tag{S7}$$

$$\left|\tau_d\right| = \left|\frac{1}{11}\sum_{i=7}^{17} \tau_i\right| \tag{S8}$$

$$\left|\tau_f\right| = \left|\frac{1}{6}\sum_{i=19}^{24} \tau_i\right| \tag{S9}$$

As mentioned in the main text, the sub-regional shear stresses are recorded every 0.1 ns of the 30-ns simulation, thus, each sub-region has a shear stress array containing 300 elements. The histogram of Figure 6a shows the distributions of elements in the shear stress array. Similarly, Figure 6b illustrates the distributions of elements in the shear stress magnitude array.

## References

[1] Cruz-Chu, E.R., Malafeev, A., Pajarskas, T., Pivkin, I.V. & Koumoutsakos, P. 2014 Structure and response to flow of the glycocalyx layer. *Biophysical journal* **106**, 232-243. (doi:10.1016/j.bpj.2013.09.060).

[2] Yamamoto, T., Ogasawara, Y., Kimura, A., Tanaka, H., Hiramatsu, O., Tsujioka, K., Lever, M.J., Parker, K.H., Jones, C.J.H., Caro, C.G., et al. 1996 Blood velocity profiles in the human renal artery by Doppler ultrasound and their relationship to atherosclerosis. *Arteriosclerosis Thrombosis and Vascular Biology* **16**, 172-177.

[3] Tufto, I. & Rofstad, E.K. 1999 Interstitial fluid pressure and capillary diameter distribution in human melanoma xenografts. *Microvascular research* **58**, 205-214.

[4] Erbel, R. & Eggebrecht, H. 2006 Aortic dimensions and the risk of dissection. *Heart* **92**, 137-142.

[5] Gabe, I.T., Gault, J.H., Ross, J., Mason, D.T., Mills, C.J., Schillingford, J.P. & Braunwald, E. 1969 Measurement of instantaneous blood flow velocity and pressure in conscious man with a catheter-tip velocity probe. *Circulation* **40**, 603-614.

[6] Hartley, C.J., Reddy, A.K., Madala, S., Entman, M.L., Michael, L.H. & Taffet, G.E. 2011 Doppler velocity measurements from large and small arteries of mice. *American Journal of Physiology-Heart and Circulatory Physiology* **301**, H269-H278.

[7] Weinbaum, S., Zhang, X., Han, Y., Vink, H. & Cowin, S.C. 2003 Mechanotransduction and flow across the endothelial glycocalyx. *Proceedings of the National Academy of Sciences of the United States of America* **100**, 7988-7995. (doi:10.1073/pnas.1332808100).

[8] Angel Gonzalez, M. & Abascal, J.L.F. 2010 The shear viscosity of rigid water models. *Journal of Chemical Physics* **132**. (doi:10.1063/1.3330544).
